# Glutamine metabolism, the Achilles heel for medulloblastoma tumor

**DOI:** 10.1038/s41419-017-0117-1

**Published:** 2018-01-22

**Authors:** Maria Victoria Niklison-Chirou

**Affiliations:** Blizard Institute, Barts and the London School of Medicine and Dentistry, Queen Mary University of London, 4 Newark Street, London, E1 2AT UK

## Body

Metabolic adaptation processes are believed to provide cancer cells with proliferation and survival benefits over normal cells. However, these processes can also make cancer cells selectively dependent, or addicted, to certain nutrients and metabolic pathways, making tumor metabolism an attractive therapeutic target^1^. One of these addictions is the dependency on glutamine (Gln). Glutamine is considered a semi-essential amino acid and, typically, in the cell it can be synthetized from the metabolism of other amino acids. Gln can be used for the cells as a source of carbon and nitrogen. As well Gln carbons can support anabolism by entering the tricarboxylic acid cycle (TCA) through glutaminolysis^2^. An increased demand for Gln by cancer cells has been recognized by researchers for almost a century^3^.

Specific tumor entities, such as MYC-overexpressing liver tumor or p73-overexpressing medulloblastoma tumors, exhibit Gln-dependency *in vivo*^4,5^. These findings are in line with glutaminase inhibitors functioning as an effective treatment for these tumors and a new generation of glutaminase inhibitors are currently being tested in clinical trials. It is commonly found that tumor cells acquire Gln-dependency as an artifact of the culture condition. Therefore, xenograft mouse models recapitulating the tumor environment must be used to claim that a tumor is “Gln-dependent”. Recently, Tardito et al. performed an extensive study concluding that glioblastoma multiforme (GBM) tumors are resistant to glutamine deprivation, which clarified recent controversial data about Gln dependency in this type of tumors. This finding could be related to the fact that, in vivo GBM tumors relies on astrocytes and glioma cells as source of Gln^6^.

Together, all these data show that to label a tumor as “Gln-dependent” we need to consider the cells of origin and the genetic variations found in the tumor as previous shown by Yuneva et al.^7^ The cell of origin is linked to the pattern of metabolic enzymes expressed by the tumor. For example, Gln and glutamate are essential amino acids playing key roles in brain metabolism and function. Indeed, in the brain, glutamine is the precursor of two neurotransmitters, glutamate and GABA, promoting the premise that brain tumors could be “Gln-dependent”.

**Fig. 1:**
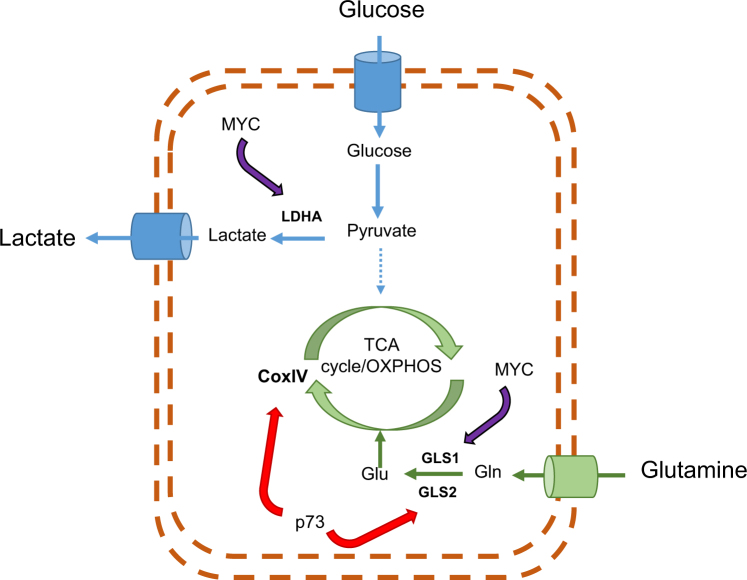
Metabolic pathways regulated by p73 or Myc in “Gln-dependent” tumors Tumor cells expressing p73 or MYC will convert most glucose to lactate and diverting glucose metabolites to support anabolic process to accelerate cell growth and proliferation. On the other hand, p73 or MYC will stimulate the use of glutamine as a source of carbon for the TCA cycle and OXPHOS. *GLS1* glutaminase 1,* GLS2* glutaminase 2, *GIn* glutamine, *Glu* glutamate, *TCA* tricarboxylic acid cycle, *OXPHOS* oxidative phosphorylation

Furthermore, the expression of different oncogenes is also linked to the cellular metabolic landscape. For example, MYC-overexpressing liver tumors will synthetize high levels of glutaminase 1 enzyme (GLS-1) and will have increased the degradation of glucose into lactate and increased glutamine consumption through the TCA, making these tumors “Gln-dependent” (Fig. 1). In contrast, MET-induced liver tumors utilize glucose to produce glutamine and therefore these tumors phenotype is glutamine independent.

Medulloblastoma (MB) are the most common malignant brain tumors in children and it is a major cause of mortality and morbidity in pediatric oncology. Current treatment include surgery, radiotherapy and chemotherapy; they have achieved 70–80% 5-year survival rates, however the treatment induces severe side effect. MB is a heterogeneous tumor which consist of at least in four distinct subgroups (Wingless [WNT], Sonic hedgehog [SHH], group 3, and group 4), with unique molecular signature, genetic mutation and clinical outcomes. Recently, it has been suggested that MB could be additionally classified based on the presence of other mutations, such as mutations in the TP53 gene. Indeed, the presence of mutant p53 in SHH-MB is associated with a poor outcome, in contrast mutation of p53 in WNT-MB is a marker of good outcome^8^.

The prognostic value of TP53 in MBs promoted several research groups to evaluate whether the other members of the p53-family could also have a prognostic value. The p53-family comprises three members: p53, p63 and p73. The role of this family in inducing apoptosis and senescence has been extensively described^9^. Unlike p53, p73 is frequently overexpressed in cancer. Accordingly, p73 is overexpressed in aggressive MB (group 3 and 4) and was shown to promote tumor cells growth and proliferation^5^.

Recently a new role for the p53-family in promoting cell survival during metabolic stress was discovered. This finding highlights on the role of p53-family in maintenance of the cellular energetic metabolism and the redox state of the cell by regulating several metabolic pathways such as glutamine metabolism (Fig. 1)^10–12^.

We investigate how p73 affect the metabolism of MB tumors by using gene manipulation and metabolomics analysis and we found that high p73 expression in MB tumor cells is associated with Gln-dependency both *in vitro* and *in vivo*. In line, we found that MB tumor cells expressing p73 exhibit high expression of glutaminase 2 (GLS-2). This is linked to MB biology as when animals were fed with a Gln-restriction diet, MB tumors growth slowed down. More importantly, Gln-restriction diet potentiated the anti-tumor activity of cisplatin (a chemotherapeutic drug commonly used for MB patient treatment), to reducing MB tumor growth and prolonging survival of treated mice. The synergism that we found between Gln-restriction diet and a chemotherapeutic agent is exciting because it brings forth a possible less toxic alternative treatment for MB patients.

The success of personalized medicine is dependent on our ability to identify the Achilles heel of a particular tumor. Indeed, “Gln-restriction diet or Gln-inhibitors” are not suitable for treatment of every tumor. However, as we increase our understanding of the particular roles that glutamine plays in tumors cells and the role played by specific genes, such as oncogenes, in glutamine metabolism, we get ever closer to developing effective targeted therapies based on the genetic landscape of the tumor.
